# The marine gastropod *Conomurex luhuanus* (Strombidae) has high-resolution spatial vision and eyes with complex retinas

**DOI:** 10.1242/jeb.243927

**Published:** 2022-08-26

**Authors:** Alison R. Irwin, Suzanne T. Williams, Daniel I. Speiser, Nicholas W. Roberts

**Affiliations:** 1 Department of Life Sciences, Natural History Museum, Cromwell Rd, London SW7 5BD, UK; 2 School of Biological Sciences, University of Bristol, 24 Tyndall Ave, Bristol BS8 1TQ, UK; 3 Department of Biological Sciences, University of South Carolina, 715 Sumter St, Columbia, SC 29208, USA

**Keywords:** Stromboidea, Conch snail, Neuroethology, Contrast sensitivity, Visual acuity, Comparative morphology

## Abstract

All species within the conch snail family Strombidae possess large camera-type eyes that are surprisingly well-developed compared with those found in most other gastropods. Although these eyes are known to be structurally complex, very little research on their visual function has been conducted. Here, we use isoluminant expanding visual stimuli to measure the spatial resolution and contrast sensitivity of a strombid, *Conomurex luhuanus*. Using these stimuli, we show that this species responds to objects as small as 1.06 deg in its visual field. We also show that *C. luhuanus* responds to Michelson contrasts of 0.07, a low contrast sensitivity between object and background. The defensive withdrawal response elicited by visual stimuli of such small angular size and low contrast suggests that conch snails may use spatial vision for the early detection of potential predators. We support these findings with morphological estimations of spatial resolution of 1.04 deg. These anatomical data therefore agree with the behavioural measures and highlight the benefits of integrating behavioural and morphological approaches in animal vision studies. Using contemporary imaging techniques [serial block-face scanning electron microscopy (SBF-SEM), in conjunction with transmission electron microscopy (TEM)], we found that *C. luhuanus* have more complex retinas, in terms of cell type diversity, than expected based on previous studies of the group using TEM alone. We find the *C. luhuanus* retina comprises six cell types, including a newly identified ganglion cell and accessory photoreceptor, rather than the previously described four cell types.

## Abstract (precis)

**Summary:** Behavioural trials indicate the eyes of conch snail *Conomurex luhuanus* provide high-resolution spatial vision, and morphological examination reveals the retina contains more cell types than those of other gastropods.

## INTRODUCTION

Eyes vary widely in form and function across the animal kingdom, with well-established associations between structure and aspects of performance. Two functional parameters often used to describe the visual performance of eyes are angular resolution (a measure of the smallest object that can be resolved by an eye) and intensity contrast sensitivity (the difference in the perceived brightness that makes an object distinguishable from its background), hereafter referred to as ‘contrast sensitivity’ (Land and Nilsson, 2012). As well as functional performance, eyes vary in complexity; generally, more complex organs comprise a greater diversity of components (McShea, 2000; Oakley and Rivera, 2008; Arendt et al., 2009). One of the most diverse animal groups in terms of visual system complexity are the Mollusca, which reflects the vast range of lifestyles in the group (Messenger, 1981; Serb and Eernisse, 2008). Within the gastropods, eye types vary from simple pits to complex camera eyes (von Salvini-Plawen and Mayr, 1977; Serb and Eernisse, 2008), yet, despite this diversity, gastropod visual systems remain relatively unexplored compared with other groups.

Species from the tropical marine family Strombidae may have eyes with the finest spatial resolution of any gastropod. Based on eye morphology, some strombids are thought to be capable of resolving objects with angular sizes as small as ∼1 deg in their visual field (Seyer, 1994). If these estimates are correct, this is surprisingly high acuity vision for a group of herbivorous gastropods. The only other gastropods with high-resolution vision are pelagic sea slugs (*Pterotracheoidea* spp.), which use their eyes to find prey (Land, 1982). Furthermore, strombid eyes may be the largest of any non-cephalopod mollusc, reaching up to ∼2 mm in diameter (Gillary and Gillary, 1979). These eyes also have a complex architecture: strombid retinas contain approximately 50,000 tightly packed photoreceptors and at least four cell types (Gillary and Gillary, 1979; Ozaki et al., 1986), and their spherical lenses appear to have a graded refractive index to reduce spherical aberration (Seyer, 1994). Moreover, electrophysiological investigations into the visual system of the strombid *Conomurex luhuanus* found evidence for multiple light responses within the retina, consistent with the presence of different types of photoreceptors, and with the occurrence of some degree of neural processing in the retina (Gillary, 1974, 1977). Together, morphological (Gillary and Gillary, 1979; Seyer, 1994) and physiological (Gillary, 1974, 1977) studies suggest that strombids have complex eyes with fine spatial resolution and high contrast sensitivity. However, predictions have yet to be verified with behavioural investigations.

In this study, we integrated behavioural and morphological approaches to explore the visual performance and retinal ultrastructure of the strombid, *C. luhuanus*. This species has been the focus of previous morphological studies on eye structure and retinal ultrastructure using traditional histological methods (Gillary and Gillary, 1979; Ozaki et al., 1986), in addition to physiological investigations (Gillary, 1974, 1977). We revisited this visual system with contemporary serial block-face SEM (SBF-SEM; Denk and Horstmann, 2004) techniques, together with TEM imaging, to classify cell types in the retina of *C. luhuanus* and discuss their possible functions. Given that functional properties of eyes are closely associated with the visual needs of their bearers (Nilsson, 2013), this combined behavioural and morphological approach of assessing spatial resolution and contrast sensitivity increases our understanding of strombid behaviour and ecology and may help to explain why these gastropods possess larger and more complex eyes than other gastropods.

## MATERIALS AND METHODS

### Sample collection

Adult *Conomurex luhuanus* (Linnaeus 1758) (*n*=20, shell length 44–52 mm; shell apex to siphonal canal) were purchased from Tropical Marine Centre (TMC), Bristol, UK and held at the University of Bristol, where they were lodged in tanks (39 litres) with seawater at a density of 4–5 conch snails per tank. Seawater in the holding system was maintained at 25–26°C and salinity 1.025–1.027 sg under a filtration system and was partially siphoned and replaced weekly to avoid accumulation of nitrates. Snails grazed on algae on the tank surfaces and within the substrate, supplemented by food pellets (TMC Gamma NutraShots *Calanus*) which were added twice weekly. Aquaria were illuminated with LED lamps under a 12 h:12 h light:dark cycle (lights on from 07:00 h to 19:00 h). Experiments commenced 1 week after the animals' arrival at the laboratory and were performed within the next 3 weeks. All experiments were conducted in accordance with the University of Bristol code of ethics for animal experimentation; approval was given by the University Animal Welfare and Ethical Review Body (AWERB) with University Investigation Number UIN/20/006. The sample size of 20 animals was chosen using G*Power v. 3.1 (Heinrich-Heine-Universität), which suggested 19 as an adequate sample size to perform the statistical tests required, with an additional animal for use in SBF-SEM studies.

### Visual behaviour experiments

#### Experimental setup

**Fig. 1.**
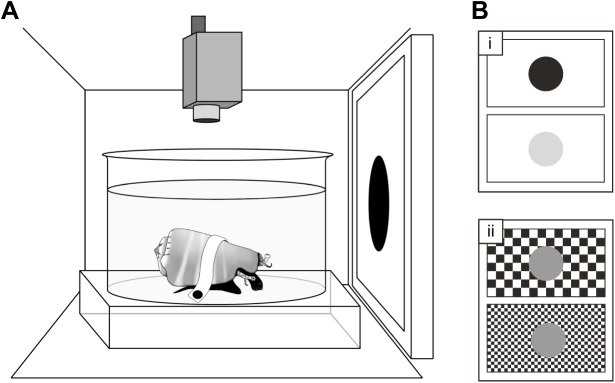
**Experimental design.** (A) *Conomurex luhuanus* was held in position within the container, placed inside a camera tent. One side of the tent was replaced with an LCD monitor (left), and behaviour was filmed from above (top). (B) Visual stimuli (expanding circles, which expand in the visual field to mimic the approach of a predator; see Movie 1) of varying (i) Michelson contrast and (ii) visual angle were presented on the monitor.

Animals were held in a glass experimental container, lightly restrained by fabric tape via magnets attached to the ends of the tape and beneath the container. The magnets allowed some freedom of movement whilst keeping the position and orientation of the snails consistent. Seawater from the holding tanks was used to fill the experimental container, with seawater changed between individuals to maintain water temperature and visibility. The experimental container was positioned in the centre of a camera tent, raised above the floor of the tent to attach the magnets (Fig. 1A). One wall of the camera tent was removed and replaced with a liquid-crystal display (LCD) monitor (Fig. 1A) on which a series of visual stimuli (described below) were displayed. This monitor, together with the camera tent, also prevented the animal from responding to slight movements around the laboratory. Animals were positioned 5 cm from the monitor and allowed to acclimatise for 10 min, or until they extended their eyestalks and proboscis and began grazing normally, encouraged by food placed inside the experimental tank. Note that the experimental container used here was cylindrical (12 cm diameter), which would produce two effects in the plane of the curvature: blurring due to spherical aberration, and reduced image size. These effects mean that it is the size of the object along the non-curved plane, rather than the curved plane, that the animal responds to; therefore, the cylindrical shape provides a limiting value on the measure of resolution.

#### Behavioural assay

Behavioural reactions by conch snails to an expanding visual stimulus (see below) were filmed from above with a digital video camera (Canon, UK) through a hole in the camera tent (Fig. 1A). Video sequences were synchronised to stimulus events using a single frequency beep produced at the start and end of each stimulus, heard only through headphones. Changes in animal behaviour before, during and after the stimulus presentations were visually identified from video playback, without knowledge of which stimuli were played in each video during scoring of animal behaviour. Behavioural changes before and during stimulus presentations were divided into seven categories to describe actions concerning the proboscis (1–3) and eyestalks (ommatophores) (4–5): (1) stop feeding; (2) partial proboscis withdrawal; (3) full proboscis withdrawal; (4) partial eyestalk withdrawal; (5) full eyestalk withdrawal (see Table 1 for full descriptions and Movie 1 for video clips of behaviours). The key change in behaviour noted in the period after stimulus presentations was the re-emergence time, defined as the time taken after the maximum response was observed for the eyestalks and the proboscis to re-emerge fully extended from the shell and for the snail to resume normal grazing behaviour.

**Table 1.**
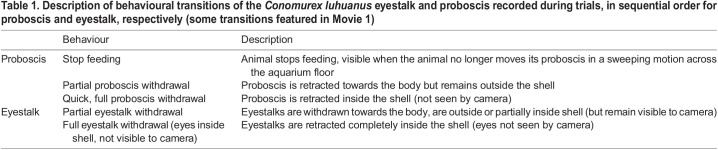
Description of behavioural transitions of the *Conomurex luhuanus* eyestalk and proboscis recorded during trials, in sequential order for proboscis and eyestalk, respectively (some transitions featured in Movie 1)

#### Expanding stimulus

Within each experiment (contrast sensitivity and spatial resolution experiments, as outlined below), a series of expanding 2-dimensional stimuli were presented in a randomised order on the monitor screen, with 3 min intervals between each stimulus presentation (or until conch snails resumed normal grazing behaviour for 3 min). Each individual was tested twice for both contrast sensitivity and spatial resolution experiments, with a rest period of at least 2 days between each of the four tests for every individual. Starting with no stimulus present on the screen background, a circular stimulus rapidly expanded (Fig. 1B; Movie 1), simulating, to the eye of the conch snails, the direct approach of a circular object with an angular size that increased from 0 deg to 83 deg of the animal's visual field. Stimuli for the contrast sensitivity and spatial resolution experiments were produced using MATLAB v. R2020a (MathWorks) or Microsoft PowerPoint, respectively. Stimuli for each experiment differed with respect to their expansion rates, as described below, so results are not directly comparable between experiments.

### Behavioural experiment 1: contrast sensitivity experiment

This experiment, following previous studies of intensity contrast using looms (e.g. Smithers et al., 2019), comprised a contrast stimulus composed of a white background with an expanding circle, with an area which enlarged at an exponentially increasing rate over a period of 10 s (Fig. 1Bi). Variation of the monitor intensity input values for the object produced nine looms with varying differences in intensity between the object and the background (pixel byte values: background=255; objects=0, 50, 100, 150, 175, 200, 210, 220 or 230), reported as Michelson contrasts (parameters close to threshold chosen based on initial observations). A grey loom on an identical grey background (Michelson contrast 0) was used as the control. The contrast threshold was determined by finding the stimulus with the lowest contrast that elicited a response.

### Behavioural experiment 2: spatial resolution experiment

This novel method comprised an isoluminant spatial resolution stimulus, composed of a black-and-white checkerboard (pixel byte values: black, 0; white, 255) background with a grey expanding circle (pixel byte value: 153). The intensity value of the grey circle was calibrated to match the mean brightness of the white and black squares in the checkerboard. When the stimulus was presented, the area of this circle enlarged at a constantly increasing rate over a period of 5 s (Fig. 1Bii). The size of the squares on the checkerboard background was varied to create eight different stimuli, with the widths of squares ranging in angular size from 0.3 to 3.2 deg. These sizes were chosen based on initial observations of *C. luhuanus* behaviour and estimates of angular resolution in conch snails from anatomical data (Gillary and Gillary, 1979; Seyer, 1994). If the eyes of conch snails were not able to resolve the black and white squares, the object and the background would appear isoluminant (of equal luminance, i.e. the mid-grey of the object) to the animal, and it should not perceive the first-order motion. Therefore, the visual acuity threshold of conch snails was determined by finding the finest checkboard (checks with smallest angular size) against which animals responded to the isoluminant expanding stimulus. As a measure of spatial resolution, we estimated the minimum resolvable angle (αmin) as twice the angular width of the smallest check to which animals responded. This experimental design used a checkerboard grating and numerous spatial frequencies, instead of a sinusoidal grating that comprises only one spatial frequency, because for this presentation the acuity required to resolve either type of contrast would be very similar. Furthermore, a sinusoidal grating introduces more opportunity for error, given the need to check every grey value displayed. Therefore, the checkerboard pattern was used for ease of programming.

### Statistical analysis of behavioural data

In calculating the probability of an individual showing a behavioural response for each stimulus type, responses where the only behavioural transition observed was ‘stop feeding’ (Table 1) were excluded to reduce the likelihood of a false positive. Wilson score intervals were calculated using the sample size for the experiment and the number of positive responses. We used Spearman's rank correlation coefficient (SRCC) to investigate whether there was a significant relationship between the response probability and Michelson contrast (contrast sensitivity experiment) or the angular size of the checks in the background (spatial resolution experiment). We also used SRCC to analyse whether there was significant correlation between re-emergence time and Michelson contrast or angular size of checks, and used paired Wilcoxon tests to explore whether there was a significant difference between the median response probability of the two repeats for each of the experiments.

We further analysed the results of these trials by using Fisher's exact test (FET) to compare the number of individuals that responded to each loom to the number that responded to the control stimulus. To account for multiple comparisons in the contrast sensitivity experiment (nine treatments and one control), we applied a Bonferroni correction.

### Serial block-face scanning electron microscopy (SBF-SEM)

#### Specimen fixation and embedding

One eye from a specimen of *C. luhuanus* was prepared for SBF-SEM work according to the following protocol. Prior to dissection, the specimen was anaesthetized in a saturated solution of 7.3% MgCl_2_·6H_2_O mixed with filtered seawater for 30 min, at which point the eyestalk withdrawal reflex was absent (Gillary and Gillary, 1979). The right eye, along with anterior parts of the eyestalk, was removed and the animal allowed to recover in a seawater tank. The sample was fixed in a 4% paraformaldehyde, 2.5% glutaraldehyde fixative in 0.1 mol l^−1^ sodium cacodylate buffer, pH 7.3, for 24 h on ice with gentle rotation to enable diffusion of the fixatives, and stored in a fresh batch of fixative at 4°C.

To prepare the sample for sectioning, a razor blade was used to slice down the centre of the eye along the sagittal plane, just off the midline (in order to section as close to the middle of the eye as possible), and one half was placed within an automated specimen preparation set-up (Leica EM TP, Leica Biosystems, UK), which ran the following steps based on the protocol of the National Center for Microscopy and Imaging Research, University of California, San Diego, CA (Deerinck et al., 2010). The tissue was washed in cold sodium cacodylate buffer (5×5 min) and fixed with a fresh solution of 1.5% potassium ferrocyanide and 2% aqueous osmium tetroxide in 0.1 mol l^−1^ cacodylate buffer for 1 h, at 4°C. At room temperature (RT) the sample was washed with diH_2_O (5×5 min), incubated with 1% thiocarbohydrazide for 20 min, and again washed with diH_2_O (5×5 min). Then, the sample was fixed in 2% osmium tetroxide in diH_2_O for 45 min, and subsequently washed with diH_2_O at RT (5×5 min). The sample was stained/fixed in 1% uranyl acetate (aqueous) overnight at 4°C and subsequently washed at RT with diH_2_O (5×5 min), and then with 0.03 mol l^−1^ aspartic acid, pH 5.5 (2×10 min). The sample was stained with Walton's lead aspartate, pH 5.5, at 60°C for 30 min and washed with 0.03 mol l^−1^ aspartic acid, pH 5.5 (2×10 min) and then diH_2_O (5×5 min), at RT. An ethanol dehydration series followed: 30%, 50%, 70% (at 4° C for 10 min each), 90% (at RT for 10 min), 100% (anhydrous; at RT for 4×15 min), propylene oxide (2×15 min). The sample was left in 1:1 propylene oxide: hard Durcupan™ mix (HDM; Sigma-Aldrich) for 1.5 h, then in 100% HDM overnight, and finally in fresh HDM for 2×3 h. Tissue was then embedded in a silicon rubber mould and polymerised at 60°C for 48 h.

#### Specimen mounting and TEM and SBF-SEM imaging

Conventional unstained sections were cut from the centre of the retina (so that images were taken from near the centre of the eye; for description of where the eye was sliced, see section above) with an ultramicrotome (Reichert Ultracut S). These sections were examined with a Tecnai T12 transmission electron microscope (Thermo Fisher Scientific UK) to obtain high-resolution TEM images of the retina and ascertain the quality of fixation prior to SBF-SEM. The resin-embedded tissue was then mounted on an aluminium specimen pin (Gatan, Pleasanton, CA, USA) using silver epoxy glue. The resin block was further trimmed with a glass knife to 1.0 mm×1.0 mm so tissue in the matrix was exposed on all four sides, with any excess silver epoxy trimmed from around the embedded tissue. The entire surface of the specimen was sputter-coated with a thin layer of gold/palladium. The block was aligned to a 3View microtome (Gatan) mounted in a Zeiss Gemini SEM 450, and a 100 nm thin sectioning of the surface begun. The block surface was imaged using BSE mode (backscattered electron detector, Gatan Onpoint detector, Gatan) over an area of 204.8 µm by 204.8 µm in the *x*–*y* plane at a resolution of 50 nm per raw pixel. The full SBF-SEM run removed 100 sections (100 nm thick), with the block face imaged after each removal.

#### Annotation and volume segmentation of retinal cells

To analyse retinal cells in a three-dimensional (3D) reconstruction, we traced a single cell through serial semi-thin sections, which is similar to procedures used to show visual structures in mice and sea spiders (Mustafi et al., 2011; Lehmann et al., 2012; Helmstaedter et al., 2013). Volume Graphics VGStudio Max v. 2.2 was used to segment retinal cells and their nuclei, pigment granules, filaments and phagosomes in 3D reconstruction. Reconstructed cells were used to measure the mean cell volume and mean total volume of pigment granules per cell via VGStudio Max, from which the density of pigment within each cell type was calculated. Counts of each cell type were made in the nuclear layer using Fiji (Schindelin et al., 2012), with cells included in the count if the cell nucleus, a key identifiable characteristic of each cell type, was visible. Estimates of the total number of each cell type per eye were calculated from cell counts within the area of retina sectioned (2.048×10^3^ mm^2^) and the total area of the retina measured previously for the same species (1.7 mm^2^; Gillary and Gillary, 1979), rounded to the nearest 100 cells.

#### Estimates of sensitivity and spatial resolution

The anatomical data was used to estimate the angular resolution of the eyes of *C. luhuanus*; note that all measurements were taken from the single eye sample used in this study and may not represent variation within the species*.* Angular resolution was calculated as twice that of the inter-receptor angle (Δφ; Land and Nilsson, 2012; Eqn 1), using the following formula:
(1)

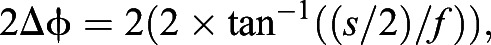
where *s* is the separation of the rhabdom centres and *f* is the focal length. Focal length was estimated as *f*=2*r* (where *r* is the radius of the lens), an assumption based on studies of lenses from strombid species *Lobatus raninus*, where the ratio of *f* to *r* was approximately 2:1 (Seyer, 1994). The absolute sensitivity of the eye under a standard luminance (*S*) was also calculated via the same method used to estimate sensitivity in the eye of *L. raninus* (Seyer, 1994), in addition to other animal groups (Kirschfeld, 1974; Land, 1981; Land and Nilsson, 2012; Eqn 2), using the following formula:
(2)

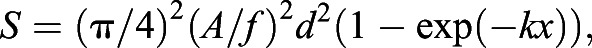
where *A* is the aperture of the eye, *d* is the rhabdom diameter, and *x* is the rhabdom length. Lastly, *k* is the absorption coefficient of the photoreceptors, 0.0067 µm^−1^, the measured value in lobster rhabdoms (Bruno et al., 1977) which was also used by Seyer (1994). While *S* is not directly comparable with contrast sensitivity behaviour experiments, this estimated value is nevertheless a useful metric in discussions of eye function.

## RESULTS

### Expanding visual stimuli elicit defensive behavioural responses from conch snails

**Fig. 2.**
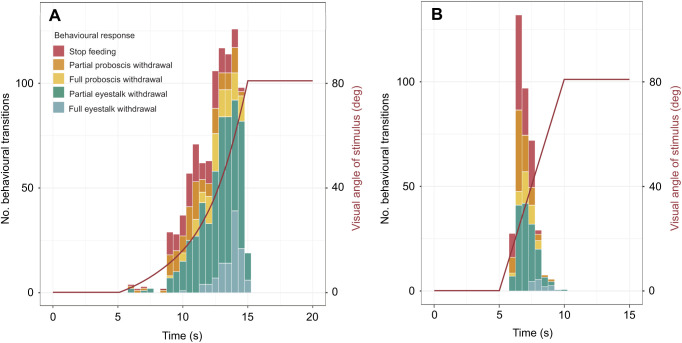
***Conomurex luhuanus* behavioural transitions displayed over the course of expanding stimulus presentations, plotted for both expanding stimulus types.** See Table 1 for full behaviour descriptions. (A) Slow stimulus (contrast sensitivity experiment, *n*=20) and (B) fast stimulus (spatial resolution experiment, *n*=19). Dark red lines represent the angular size of the stimuli (right *y*-axis, log scale), which increases (A) exponentially or (B) linearly with time. Each experiment includes two replicates for every animal used.

*Conomurex luhuanus* responded to expanding visual stimuli with a series of defensive behaviours involving retraction of the ommatophores and proboscis (Table 1). These are distinguishable from normal grazing activity where the ommatophores and proboscis are extended, the latter moving constantly in a searching motion to feed (Movie 1). Behavioural responses (Fig. 2) are more easily separable into a sequence when visual stimuli expand relatively slowly (Fig. 2A) rather than relatively quickly (Fig. 2B). Over the expansion period of the stimulus, the following behaviours were observed, in sequential order: animals stopped feeding; proboscis and eyestalks were partially withdrawn towards the body (remaining outside the shell; Table 1; Fig. 2A; Movie 1); eyestalks and proboscis were fully retracted inside the shell and no longer seen by the camera (Table 1; Fig. 2A; Movie 1).

Animals became more likely to exhibit defensive behaviours during the rapid expansion phase of the expanding circle; after the stimulus had subtended 37.9 deg of the visual field, 62% of total behavioural transitions in this experiment and 92% of full eyestalk withdrawals were recorded (Fig. 2A). Only one animal fully withdrew its eyes before the stimulus reached 27.5 deg in the visual field (Fig. 2A). Fewest responses (0.01% of total behavioural transitions) were recorded during the slow expansion phase of the expanding circle, when the stimulus had subtended 2.3–9.8 deg of the visual field (Fig. 2A). During this time, only initial changes in behaviour were recorded (stop feeding, partial withdrawal of proboscis and eyestalk towards the shell; Table 1; Movie 1).

### *Conomurex luhuanus* responds to Michelson contrasts of 0.07

**Fig. 3.**
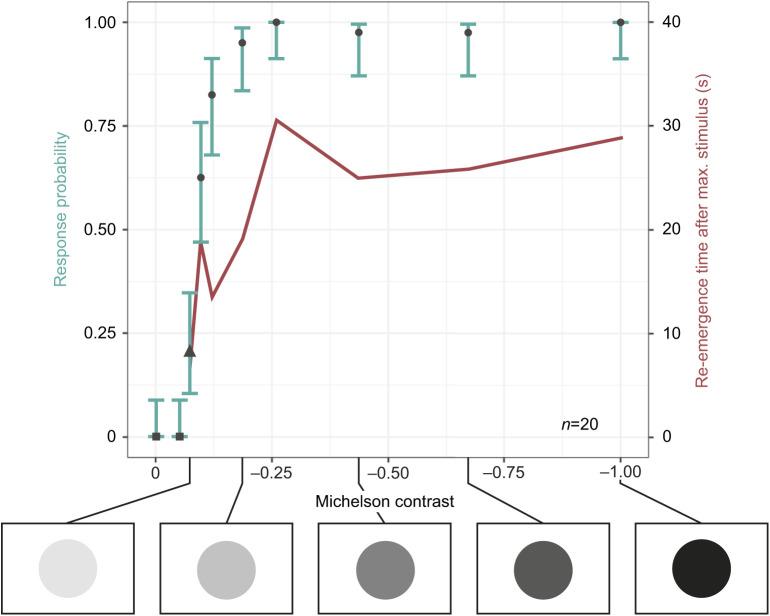
**Response probabilities of *C. luhuanus* to expanding stimuli varying in Michelson contrast.** Square, *P*=1; triangle, *P*≤0.05; circle, *P*≤0.01 following Bonferroni correction (*n*=40, i.e. 20 individuals tested twice). Error bars (green) are Wilson score intervals. Re-emergence time (red) is the time taken post-stimulus for ommatophores to return to their extended position and normal grazing to resume. This experiment included two replicates for every animal used.

The probability of a conch snail showing a behavioural response to a looming visual stimulus is positively correlated with the magnitude of the Michelson contrast of the stimulus (SRCC, ρ=0.942, *n*=40, *P*<0.001; Fig. 3). In this experiment, 20% of individuals responded to looming visual stimuli with a Michelson contrast of 0.07 (FET, *n*=40, *P=*0.002) and 62.5% of individuals responded to those with a Michelson contrast of 0.10 (FET, *n*=40, *P<*0.001) (Fig. 3). The re-emergence time also showed a strong positive correlation with Michelson contrast magnitude (SRCC, ρ*=*0.883, *n*=40, *P*=0.003; Fig. 3). There was no significant difference in the median response probability between the two repeats of this experiment (Wilcoxon, *V*=12, *n*=20, *P*=0.281).

### *Conomurex luhuanus* has a spatial resolution of 1.06 deg

**Fig. 4.**
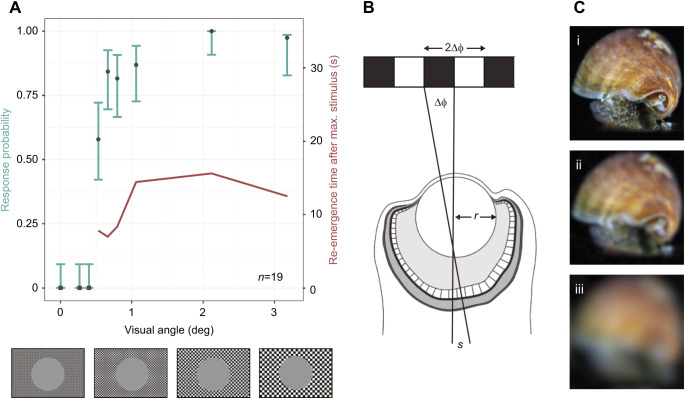
**Calculation of visual acuity of *Conomurex luhuanus* based on behaviour and morphological data, with depiction of acuity.** (A) Response probabilities (black shapes) of *C. luhuanus* to expanding stimuli with visual angle of checkerboard background squares varied (below, not to scale): square, *P*=1; circle, *P*≤0.01 following Bonferroni correction (*n*=38, i.e. 19 individuals tested twice). Error bars (green) are Wilson score intervals. Re-emergence time (red) is the time taken post-stimulus for ommatophores to return to their extended position and normal grazing to resume. This experiment included two replicates for every animal used. (B) Diagram illustrating the finest checkerboard that the eye can resolve, with an angular period of twice the inter-receptor angle Δφ. (C) Image of *C. luhuanus* blurred using R v.4.0.3 via the package AcuityView (Caves and Johnsen, 2018), according to the spatial resolution of: (i) octopus (Land, 1981), (ii) *C. luhuanus* (this study), (iii) periwinkle *Littorina littorea* (Seyer, 1992).

Animals responded to an expanding isoluminant stimulus against a black and white checkerboard pattern consisting of checks with angular widths of 0.53 deg (FET, *n*=38, *P*<0.001) and greater (Fig. 4A). From this response, the minimum resolvable angle of *C. luhuanus* was 1.06 deg, twice the angular width of the narrowest square checks against which it responded to looming isoluminant stimuli. The probability of an individual showing a behavioural response was positively correlated with the angular sizes of the checks in the checkerboard background (SRCC, ρ*=*0.928, *n*=38, *P*<0.001; Fig. 4A). The re-emergence time showed a positive correlation with the angular sizes of the checks (SRCC, ρ*=*0.898, *n*=38, *P*=0.002; Fig. 4A). There was no significant difference in the median response probability between the two repeats of this experiment (Wilcoxon*, V*=4, *n*=19, *P*=0.419).

### Eye anatomy suggests fine spatial resolution and high sensitivity in *C. luhuanus*

Spatial resolution and sensitivity were estimated from the single *C. luhuanus* eye used for SBF-SEM, using average values for *s*, *x* and *d* [Eqns 1 and 2; expressed as: means±s.d. (s.e.m.; *n*, number of measurements taken from the eye)]. The error values provide a measure of the variance within the eye. The spatial resolution estimated from anatomical data [*s*=6.5±0.9 µm (s.e.m., 0.1 µm; *n*=56); *f*=720 µm] was calculated as 1.04 deg, twice the interceptor angle of 0.52 deg (Fig. 4B; Eqn 1). The sensitivity value *S* of the *C. luhuanus* eye was calculated to be 7.78 µm^2^ sr using anatomical data [*A*=630; *f*=720 µm; *d*=6.6 µm±0.8 (s.e.m., 0.1 µm; *n*=68); *x*= 70.9±2.7 µm (s.e.m., 0.6 µm; *n*=20)] (Eqn 2).

### The retina of *C. luhuanus* contains at least six cell types

**Fig. 5.**
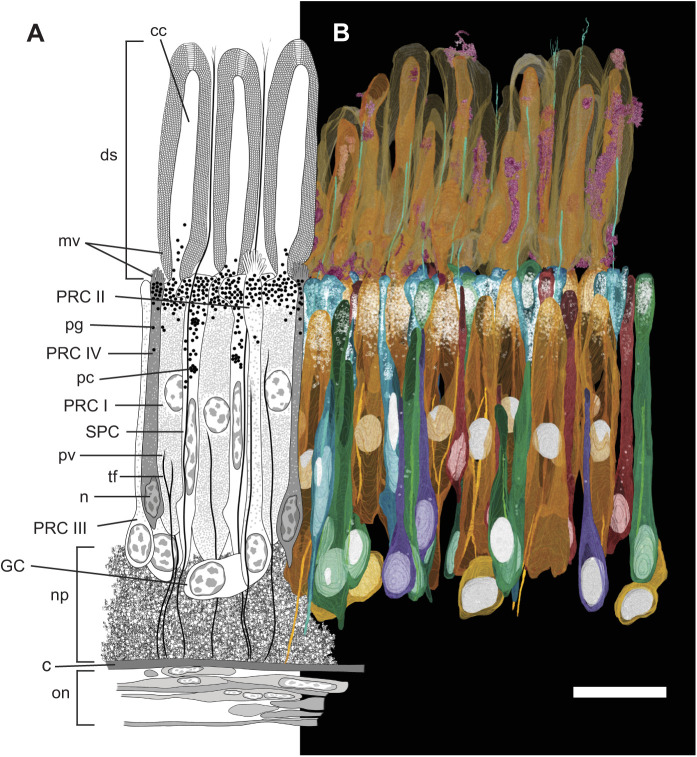
**Retina structure of *Conomurex luhuanus*.** (A) Diagram (cell bodies and nuclei to scale) representing the supportive cell (SPC), ganglion cell (GC) and photoreceptor cells (PRC I–IV). (B) Segmentation and reconstruction of cells using SBF-SEM data: blue, SPC; orange, PRC I; green, PRC II; purple, PRC III; red, PRC IV; yellow, GC; pink, phagocytic activity. Nuclei and pigment are highlighted in white. Abbreviations: c, capsule; cc, cytoplasmic core (of PRC I distal segment); ds, distal segments; mv, microvilli; n, nucleus; np, neuropile; on, optic nerve; pc, pigment cluster; pg, pigment granule; pv, photic vesicles; tf, tonofilaments. Scale bar: 20 µm. Note that following reconstruction, the individual cells in B have been layered consecutively using Adobe Photoshop v. 22.5.3 (Adobe Inc.) so that they are in the right position and overlying or underlying the correct adjacent cells. In this way, the semi-transparency of the cells, used in order to display pigment and nuclei, does not interfere with the clarity of the image (for unedited image, see Fig. S1). To ensure the image remained representative of the data, positioning of the cells was verified using an underlying layer of the whole reconstructed retina for reference, since removed. All data are from a single eye of one specimen.

**Fig. 6.**
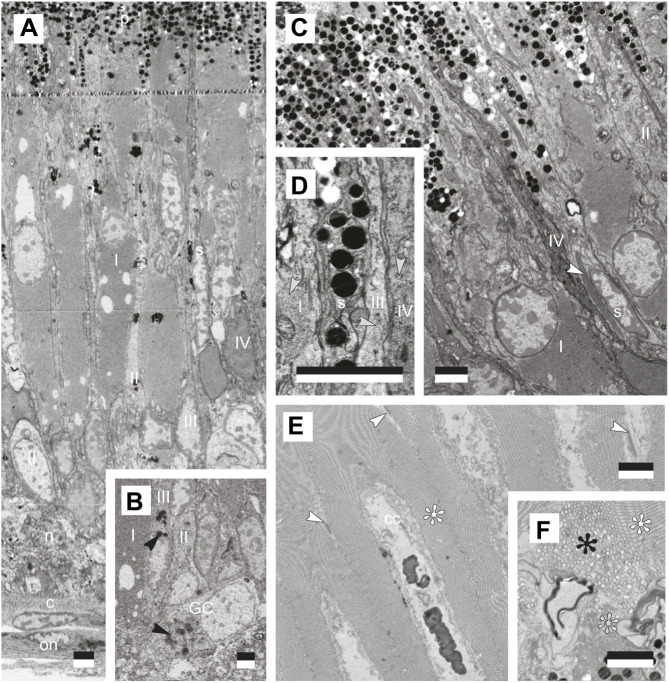
**Structural characterisation of *Conomurex luhuanus* supportive (SPC) and photoreceptor retinal cells (PRC I–IV).** TEM (A,C–F) and SBF-SEM (B) images show: (A) cells within the nuclear region; (B) cells close to the neural layer, with GC and PRC III containing large dense bodies (black arrows); (C) cells at the nuclear and pigmented regions; (D) presence of photic vesicles in cells PRC I, III and IV (grey arrows) and absence in SPC; (E) distal segments of PRC (I; F) microvilli of PRC IV (grey asterisk) and PRC II (black asterisk). For a close-up of photic vesicles within the same species, see fig. 5 in Gillary and Gillary (1979). Abbreviations: c, capsule; cc, cytoplasmic core (of PRC I distal segment); n, neuropile; on, optic nerve; p, pigmented region; s, SPC; I–IV, PRC I–IV. White arrows: tonofilament; white asterisk: PRC II microvilli. Scale bars: 2.5 µm. All data are from a single eye of one specimen.

The large *C. luhuanus* eye (diameter 1.2 mm, excluding eyestalk tissue) contains a retina comprising several broad layers contained within the capsule: a distal segment layer, pigmented region, nuclear layer and neuropile (Gillary and Gillary, 1979; Fig. 5). The area of the retina sectioned by SBF-SEM data (2.048×10^3^ mm^2^) contained 189 cells in the nuclear layer, from which an estimate of 1.57×10^5^ total retinal cells per eye was produced (Table 2). Cell types were distinguishable by differences in outer and inner segment morphology, inner segment electron density, and nucleus morphology and position, as well as various other cellular inclusions as described in the following sections. Finer cellular features such as axons were not discernible at the resolution of the SBF-SEM data. Note that photic vesicles are identified via TEM based on comparisons with other gastropod studies, which discuss their role in photoreception (Eakin and Brandenburger, 1975; Ozaki et al., 1986; Eakin, 1990); see Discussion. With SBF-SEM data, we could readily discern six morphologically distinct retinal cells, clearly separable in the nuclear layer: a supportive cell (SPC), four photoreceptor cell types (PRC I–IV) and a ganglion cell (GC) (Figs 5 and 6; Table 2; for comparisons of cells with previous studies, including axons, see Discussion). Cell types PRC IV and GC are newly described within the *C. luhuanus* retina from this study.

**Table 2.**
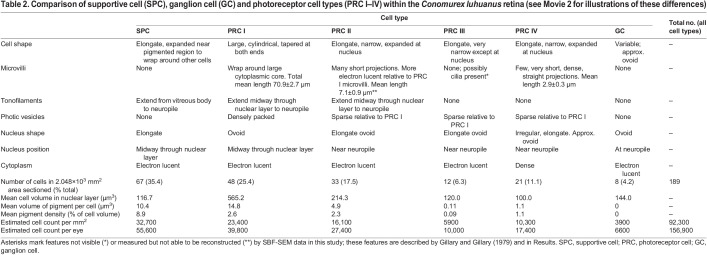
Comparison of supportive cell (SPC), ganglion cell (GC) and photoreceptor cell types (PRC I–IV) within the *Conomurex luhuanus* retina (see Movie 2 for illustrations of these differences)

As sectioning did not occur precisely down the midline of the eye, photoreceptors were at a slight angle, and complete outer and inner sections could not be entirely traced. Some intracellular features were also not traced to completion, or were not detected in the sample sectioned, such as the thin bundles of tonofilaments (Fig. 5B; see Movie 2 for reconstructions). Thus, photoreceptors were traced as near to completion as possible (Fig. 5B; Movie 2), and a diagram produced based on these and previous findings to illustrate cell structures as described below (Fig. 5A).

#### Supportive cell (SPC)

**Fig. 7.**
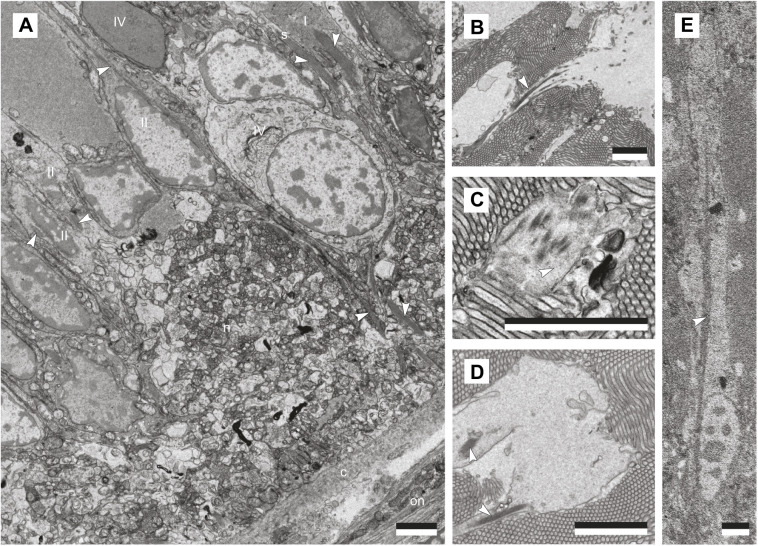
**Morphological characterisation of filaments within *Conomurex luhuanus* retinal cells.** TEM images show: (A) bundles of filaments in supportive cells (SPC) and photoreceptor cells (PRC I–II) within the nuclear and neuropile layers; (B) SPC tonofilament dispersing into vitreous body; (C) SPC tonofilaments dispersing within the distal segment layer; (D) cytoplasm and membrane not always tightly bound around SPC tonofilament. (E) SBF-SEM image shows PRC II filaments extending midway through the nuclear layer before dispersing. Abbreviations: c, capsule; n, neuropile; on, optic nerve; s, SPC; I–IV, PRC I–IV. Scale bars: 2.5 µm. All data are from a single eye of one specimen. White arrows indicate filaments.

Of the six cell types observed in the *C. luhuanus* retina (Fig. 5 and Fig. 6A–C), supportive cell (SPC) types are the most abundant (35.4% of total cells; Table 2). These cells are electron-lucent, lacking photic vesicles (Table 2; Fig. 5 and Fig. 6A,C,D). The nuclei are narrow, elongated, and vary with respect to their positions within the nuclear layer (Fig. 5 and Fig. 6A,C; Movie 2). The cell body is narrow, except within the pigmented region, where it expands to surround adjacent photoreceptor cells (Fig. 5; Movie 2). SPC is the most heavily pigmented retinal cell type (8.9% of the total cell volume; Table 2); unlike other cell types, pigment granules extend from the pigmented region of the retina as far as the nucleus (Fig. 5 and Fig. 6A,C,D; Movie 2). SPC pigment granules sometimes form clusters within the nuclear layer, bound by a membrane (Fig. 5 and Fig. 6D; Movie 2). A bundle of densely packed tonofilaments extends from the capsule (Fig. 7A), through the nuclear layer and between the photoreceptor cell type I distal segments in the rhabdomeric layer (Fig. 6C,E and Fig. 7D), finally dispersing in the vitreous body, or else prematurely between the distal segments (Fig. 7B,C).

#### Photoreceptor cell type I (PRC I)

The main, most abundant (Table 2) photoreceptor cell, PRC I, possesses long (70.9±2.7 µm) distal segments that together comprise the majority of the rhabdomeric layer (Fig. 5 and Fig. 6D; Movie 2). The cell body of PRC I in the nuclear layer is wide, tapering in the pigmented region and towards the neuropile, and contains ovoid nuclei positioned midway through the nuclear layer (Fig. 5 and Fig. 6A–C; Movie 2). The cytoplasm is packed with spherical vesicles, identified as photic vesicles (Fig. 6D), also containing bundles of filaments extending from the basal end of the retina to midway through the nuclear layer (Fig. 5 and Fig. 7A; Movie 2), although these filament bundles were rarely identified in the cell. In the distal segment layer, each long photosensitive organelle consists of a central cytoplasmic shaft extending out from the cell body, with an array of microvilli projecting from the surface, circularly curved around the central shaft (Fig. 5 and Fig. 6D; Movie 2; see also Gillary and Gillary, 1979).

#### Photoreceptor cell type II (PRC II)

PRC II is less abundant than PRC I, with many short [7.1±0.9 µm (s.e.m., 0.2 µm; *n*=20)] microvilli extending from the apical end of the cell body, instead of from a cytoplasmic core as in PRC I (Fig. 5A and Fig. 6E; Movie 2). These microvilli are more electron lucent and disordered compared with the regularly arranged microvilli in the longer PRC I distal segments (Fig. 6F). Owing to the plane in which the retina was sectioned, microvilli in these SBF-SEM data could not be accurately reconstructed. The cytoplasm is electron lucent and contains sparse photic vesicles, with a subspherical nucleus close to the neuropile (Figs 5–7; Movie 2). The cytoplasm contains bundles of filaments which extend from the neuropile to midway through the nuclear layer (Fig. 5 and Fig. 7A,E; Movie 2).

#### Photoreceptor cell type III (PRC III)

PRC III is found infrequently within the retina and possesses a very narrow soma with electron-lucent cytoplasm, and a nucleus located close to the neuropile (Table 2; Figs 5–7; Movie 2). TEM images show sparse photic vesicles scattered in the cytoplasm (Fig. 6D), with large dense bodies identified in SBF-SEM sections (Fig. 6B). SBF-SEM data also revealed a lack of screening pigment in the pigmented region, with sparse granules scattered in the nuclear layer (Table 2; Fig. 5; Movie 2).

#### Photoreceptor cell type IV (PRC IV)

This cell type has not previously been observed in morphological studies of strombid retinas. PRC IV possesses very few microvilli projecting into the distal segment layer from a flat, apical surface (Fig. 5 and Fig. 6F). These microvilli are much shorter than those of PRC II and PRC III [2.9±0.3 µm (s.e.m., 0.1 µm; *n*=12)], and, like the remainder of the cell cytoplasm, are very electron dense compared with all other cell types (Fig. 6 and Fig. 7A). The cell body is of a similar overall shape to that of PRC II: narrow in the pigmented region and much of the nuclear layer, widening at its irregularly shaped nucleus near the neuropile (Figs 5–7; Movie 2).

#### Ganglion cell (GCs)

Like PRC IV, GCs have not previously been identified in strombid retinas and is the least frequent component of the retina (Table 2). This ganglion cell is variable in shape and size but is approximately ovoid, with a large, ovoid nucleus (Fig. 5 and Fig. 6B). The cytoplasm is electron lucent and lacks photic vesicles, instead containing numerous large dense bodies (Fig. 6B).

#### Phagosomes

**Fig. 8.**
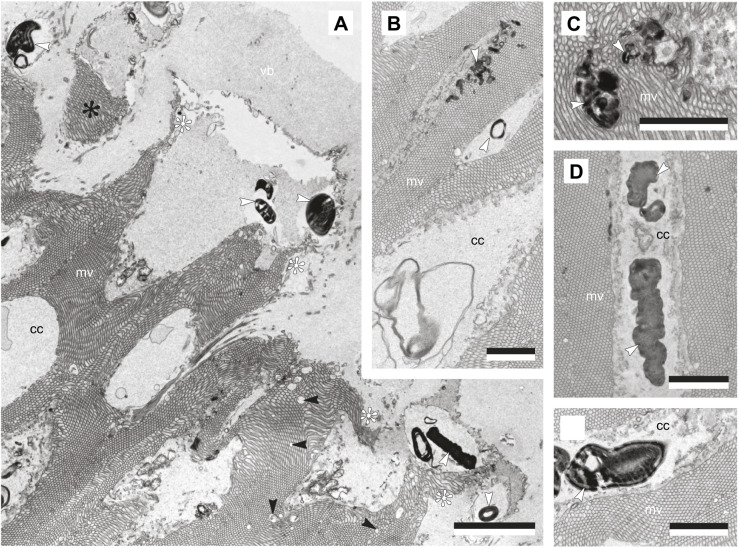
**Morphological characterisation of phagosomes within the main *Conomurex luhuanus* photoreceptor cell (PRC I) distal segment layer.** TEM images show: (A) phagosomes (white arrows) at apical end of distal segments, with punctuate discontinuities in the microvillar array (black arrow), and apical ends of distal segments curling away (white asterisk) or completely separated (black asterisk) from rest of distal segments; (B,C) phagosomes (white arrows) intruding on microvillar arrays; (D,E) phagosomes (white arrows) within cytoplasmic core of distal segments. Abbreviations: cc, cytoplasmic core; mv, microvilli; vb, vitreous body. Scale bars: 2.5 µm. All data are from a single eye of one specimen.

SBF-SEM and TEM data were also used to identify electron-dense, lamellar inclusions in the distal segment layer of the retina, identified as phagosomes (Fig. 5B and Fig. 8). Their structures, albeit diverse, all comprise concentric systems of membranes, mostly irregular in shape, with few circular types (e.g. Fig. 8A). They have no specific intracellular location within the cytoplasmic core and can be found in the basal or apical portions (Fig. 5 and Fig. 8B–E), with others located within vacuole-like structures just above the apical end of the distal segments, within the vitreous body (Fig. 8A; Movie 2). Some are located between the main distal segments, surrounding the microvilli projecting from the accessory photoreceptor cells (Fig. 5B; Movie 2).

## DISCUSSION

This is the first study to link visually influenced behaviours in conch snails to the structure and function of their well-developed eyes. In this study, the use of behavioural experiments together with volumetric electron microscopy has enabled new insights into the strombid visual system, the findings of which are discussed below.

### Strombids have high spatial resolution

Estimates of spatial resolution from behavioural (αmin=1.06 deg) and anatomical (2Δφ=1.04 deg) measurements both suggest that the strombid eye can resolve objects of ∼1 deg in the visual field (Fig. 4). These estimates of visual acuity from the anatomy of the *C. luhuanus* eye mirror previous anatomical estimates from another strombid species, *Lobatus raninus* (2Δφ=0.94 deg, based on multiple specimens; Seyer, 1994). In both species, resolution may be coarsened by a lack of pigment shielding between rhabdoms, which would result in retinal spread (Seyer, 1994); however, lack of pigment shielding does not appear to impact our anatomical estimate of acuity, given the close match between estimates from behavioural and anatomical data. Thus, while the potential for high resolution strombid vision was suggested by Seyer (1994), the combined behavioural and anatomical approaches used in this study provide conclusive evidence that these animals utilize this resolution in visual tasks. A spatial resolution of 1 deg in the strombids is much finer than is estimated for other gastropods; these vary from 3.6 deg in *Littorina littorea* (anatomical data; Seyer, 1992), to 52 deg in *Arion rufus* (behavioural data; Zieger et al., 2009). Like the strombids, these are mostly non-predatory gastropods. Apart from the predatory heteropods, with a spatial resolution of 0.41 deg, the only other molluscs with fine-resolution vision are cephalopods, which use their high acuity to target prey (Land, 1981, 1982; Gagnon et al., 2013). Cephalopod eyes range widely in resolution, from 0.57 deg in the cuttlefish *Sepia officinalis* (Groeger et al., 2005) and 0.50 deg in the squid *Japetella* sp. (Sweeney et al., 2007) to 0.04 deg in *Octopus vulgaris* (Young, 1962a).

The ability of *C. luhuanus* to utilize high resolution information is also indicated by the high density of main photoreceptor cells in its retina (PRC I), estimated to be 3.98×10^4^ per eye (Table 2), although this does not consider the possible variation in cell density across the retina, as noted for cephalopods (Young, 1962a). This estimate of PRC I cells is less than the 5×10^4^ PRC I cells suggested by Gillary and Gillary (1979), and closely matches the 4×10^4^ optic nerve fibres estimated from the same study. Nevertheless, both estimates of PRC I suggest that more numerous photoreceptors are present in the eyes of conch than in other gastropods, such as *Cornu* and *Onchidium* spp., with 2500–3800 and 600 main photoreceptor cells per eye, respectively (Brandenburger, 1975; Katagiri et al., 1995); by comparison, *O. vulgaris* is estimated to possess 2.0×10^7^ photoreceptors per eye (Young, 1962a). Hess (1905) estimates the photoreceptor density in *S. officinalis* to be 105,000 per mm^2^; however, in *O. vulgaris*, the estimated density (70,000 per mm^2^) is not dissimilar to that within *C. luhuanus* (55,700 per mm^2^ for PRC I–IV or 23,400 per mm^2^ for PRC I only), although in *O. vulgaris*, 25% of the cells in the nuclear layer are supporting cells, as opposed to 37% in *C. luhuanus* (excluding ganglion cells; Table 2; Young, 1962a).

Spatial resolution is one of several variables that determine the visual tasks an eye can support. In particular, it is an important property for detecting moving objects in the environment, especially potential predators (Nilsson, 2013). Like other strombids, *C. luhuanus* are sometimes found in large aggregations of 100–200 individuals, between <1 and 30 individuals per m^2^; an easy target for some visual predators (Poiner and Catterall, 1988; Ulm et al., 2019). While their leaping escape behaviour may help strombids avoid slow-moving predators such as cone snails (Berg, 1974, 1975; Field, 1977), this escape response is too slow to be effective against fast-moving predators such as fish, shell-peeling crabs or octopuses (Savazzi, 1991). Therefore, strombids must detect approaching objects and withdraw into their shell, relying on its passive mechanical protection (Savazzi, 1991), though the shell can be crushed by certain predators (Berg, 1974).

Behavioural experiments demonstrated that an expanding circle subtending 2.3–9.8 deg of the visual field could cause *C. luhuanus* to stop feeding and slowly withdraw their eyestalks and proboscis; i.e. a larger size than the ∼1 deg that anatomical and behavioural estimates of spatial resolution suggest they can resolve. However, additional defensive responses only occurred after the stimulus expanded to an angular size of 11.8 deg (Fig. 2A). This possibility indicates two different behavioural thresholds in response to approaching objects: firstly, the point at which the snail ceases other activity to focus on the approaching object; secondly, the point at which the snail actively avoids a predator. This suggests that one function of high visual acuity in strombids is to detect potential predators as early as possible. Behavioural studies of other non-predatory molluscs with poorer spatial resolution have demonstrated a variety of uses of visual information: detecting potential predators [e.g. bivalve *Cardium edule* (Barber and Wright, 1969) and scallop *Argopecten irradians* (Chappell et al., 2021)], orienting to celestial cues and finding suitable habitats (e.g. *Littorina* sp.; Fig. 4C; Newell, 1958; Hamilton, 1977). Therefore, previous authors have suggested that vision in strombids may also support other behavioural tasks, such as the escape response triggered by the presence of molluscivorous cone snails (Field, 1977), striking a predator more accurately with a kick of its long, serrated operculum as a deterrent (Prince, 1955; but see Berg, 1974), or finding conspecifics and suitable habitats. However, the role of spatial vision in these behaviours has yet to be tested.

### Strombids have both high contrast sensitivity and absolute sensitivity

In addition to high spatial resolution, high contrast sensitivity is also advantageous for early predator detection. Experiments with the expanding visual stimuli showed *C. luhuanus* to be capable of discriminating between small differences in light intensity (Michelson contrast 0.07; Fig. 3). Within strombids, the ability to detect small changes in contrast within any environment allows for a larger, safer distance at which potential predators are identified (Land, 1981; Smolka and Hemmi, 2009). By comparison, fixation reflex experiments in the cephalopod *Octopus tetricus* suggested that animals responded to differences in contrast of 1–4% (equivalent to a Michelson contrast of 0.005–0.02; Nahmad-Rohen and Vorobyev, 2019).

Regarding the absolute sensitivity of the eye, increased photon capture is facilitated through several adaptations seen in strombid eyes (Hughes, 1976; Gillary and Gillary, 1979; Seyer, 1994), including wide apertures to allow more light into the eye, long distal segments of the photoreceptors to increase the absorbance path length, and photoreceptors with wide acceptance angles (Land and Nilsson, 2012). The absolute sensitivity value, *S* of the *C. luhuanus* eye (7.78 µm^2^ sr), is less than values previously calculated for the strombid *Lobatus raninus* (9.9 µm^2^ sr; Seyer, 1994); differences may be accounted for by the large variation in rhabdom length in the retina (Gillary, 1974, plate 1; our observations), meaning that measurements taken from the area sectioned in this study may not be representative of the sensitivity of the whole eye (for description of the area sectioned, see Materials and Methods). These values are nevertheless similar to those calculated for octopuses (9.7 µm^2^ sr; Hanlon and Messenger, 2018), which often inhabit similar coastal sea floor environments to *C. luhuanus* (predominantly sand, coral rubble and seagrass beds; Poiner and Catterall, 1988; Ulm et al., 2019).

Sensitivity estimates from morphological data may also reflect the fact that some strombid species appear to be most active in dim light [*L. raninus* (Seyer, 1994) and *C. luhuanus* (our observations)], although laboratory and field studies of strombids have observed some feeding activity around the clock (Randall, 1964; our observations). Comparisons between the eyes of known nocturnal and diurnal species suggest that strombid *S* values are congruous with twilight activity (Seyer, 1994; Land and Nilsson, 2012), a prediction supported by features of the strombid eye that make it highly sensitive to light. These include the long length of the PCR I distal segments (mean 70.9±2.7 µm; Fig. 5) and their specialised structure: a cytoplasmic core extending from the pigmented region of the cell, around which arrays of microvilli are circularly arranged (Fig. 6D; Hughes, 1976; Gillary and Gillary, 1979). The cytoplasmic core is suggested as an adaptation for more efficient transport of materials in longer distal segments (Hughes, 1976); however, this structure may also increase sensitivity due to microvillar orientation. In both the circular arrays of microvilli in strombids and the brush-like arrays in gastropods *Bulla*, *Limax*, *Deroceras* and *Athoracophorus* spp. (Katoaka, 1975; Eakin et al., 1980; Jacklet and Colquhoun, 1983; Newell and Newell, 1968), microvilli are oriented perpendicular to the incident light entering the eye, allowing maximum absorption of photons and thereby increasing visual sensitivity (Eakin et al., 1980). This is consistent with the fact that species in all these genera are nocturnal (Carmichael, 1931; Stephenson, 1968; Eakin et al., 1980; Jacklet and Colquhoun, 1983), and that strombids are also observed to be active in dim light.

### The strombid retina is composed of at least six different cell types

If a measure for organ-level complexity is the diversity of parts out of which a given organ is composed (McShea, 2000; Oakley and Rivera, 2008; Arendt et al., 2009), the six cell types within the *C. luhuanus* retina indicates that conch have more complex eyes than any other gastropod studied to date. In contrast to the six retinal cells (4 photoreceptors, 1 ganglion cell and 1 supportive cell) identified in *C. luhuanus* (Table 2; Figs 5–7; Movie 2), most gastropods possess only two or three retinal cell types: a supportive cell, a main photoreceptor cell, and sometimes an accessory photoreceptor cell with shorter distal segments (e.g. Eakin et al., 1967; Jacklet and Colquhoun, 1983; Seyer, 1992; Seyer et al.,1998; Pinchuck and Hodgson, 2018). Despite belonging in the same superfamily as the strombid *C. luhuanus*, only two cell types are identified in aporrhaid *Aporrhais pespelecani* (Blumer, 1996)*.* An exception to this is seen in the retinas of *Cornu aspersum* and *Onchidium verruculatum*, wherein a fourth (Brandenburger, 1975) or fifth (including ganglion cell; Katagiri et al., 1995) cell type was identified but not described with detail owing to low frequency in the retina. These comparisons indicate a remarkably complex visual system in *C. luhuanus* compared with those known for most other gastropods. However, the above studies were all performed using TEM only, as opposed to TEM with SBF-SEM as in this study. The fact that more cells were found within the conch snail retina in this study than in previous studies of the same species (e.g. Gillary and Gillary, 1979) suggests the possibility of a higher complexity in other gastropod retinas too, and should be revisited with contemporary methods.

Previous studies using TEM alone suggest that the *C. luhuanus* retina contains four different cell types: a supportive cell (SPC) and three types of photoreceptors (PRC I–III) (Gillary and Gillary, 1979; Ozaki et al., 1986). Histological studies in other strombid species identified only two of these cells, SPC and PRC I (Prince, 1955; Hughes, 1976). Of the six cell types identified in this study using SBF-SEM and TEM data, two are newly identified (PRC IV and GC) and four matched those described in *C. luhuanus* in previous studies (Gillary and Gillary, 1979; Ozaki et al., 1986; Table 2). However, PRC III is only putatively identified because of the limited description in Gillary and Gillary (1979) and differences in imaging resolution between data in this and previous studies. Nevertheless, the possible fourth cell described by Gillary and Gillary (1979) and PRC III share a very narrow soma, electron-lucent cytoplasm, infrequent occurrence, and position of the nucleus close to the neuropile, indicating a strong likelihood that these are the same cell type (Table 2; Figs 5–7; Movie 2). Furthermore, SBF-SEM data did not identify microvilli at the apical end of PRC III, consistent with previous work (Gillary and Gillary, 1979; Ozaki et al., 1986).

Despite several differences, these retinal cells share key features with those reported for other gastropod species, allowing discussion of the likely function of the cell types described in this study (Figs 5–7). Unlike supportive cells, gastropod photoreceptor cells contain many small electron-lucent cytoplasmic vesicles, referred to as photic vesicles (e.g. Eakin et al., 1967; Eakin and Brandenburger, 1975; Stoll, 1973; Hughes, 1976; Gillary and Gillary, 1979; Gibson, 1984; Eakin, 1990; Pinchuck and Hodgson, 2018). The distribution of these vesicles varies across groups and between cell types; in cells such as PRC I in *C. luhuanus*, photic vesicles are densely packed throughout the cytoplasm (e.g. Pinchuck and Hodgson, 2018; Fig. 6D), whereas investigations within slugs revealed aggregations just beneath the light-sensitive microvilli in the light-tolerant *Ariolimax* sp. or concentrated basally near the nuclei in the nocturnal *Limax* sp., supporting the suggestion that the vesicles are associated with photoreception (Eakin and Brandenburger, 1975). Previous studies suggest several functions for these vesicles, including storage of photopigment (Röhlich and Török, 1963; Eakin and Brandenburger, 1968; Eakin, 1990). Within *C. luhuanus* (Ozaki et al., 1986) and *Onchidium* sp. (Katagiri et al., 2001), an abundance of the photopigment retinochrome was found in fractions of photoreceptor cells containing photic vesicles, supporting the idea that these are involved in storage. The presence of these vesicles in PRC I–IV within *C. luhuanus* (albeit more sparsely in PRC II–IV; Fig. 6D; see fig. 5 in Gillary and Gillary, 1979 for a close-up of photic vesicles in the same species) therefore indicates that these cells are involved in photoreception.

Unlike the photoreceptor cells, the newly described ganglion cell in this study lacks photic vesicles, instead containing numerous large dense bodies, likely secondary residual lysosomes as identified in other gastropod studies (Fig. 6B; Eakin et al., 1980). This cell is analogous to a variety of cells located exclusively in the neural layer of the retina in previous gastropod studies, described as ganglion cells, secondary cells and neurosecretory cells or neurons; see these examples for ganglion cell axons, which could not be identified at the resolution of SBF-SEM data in this study (Fig. 5 and Fig. 6B; Stoll, 1973; Brandenburger, 1975; Eakin et al., 1980; Jacklet and Colquhoun, 1983; Katagiri et al., 1995). The newly described cell PRC IV differs predominantly from other retinal cells in that its cytoplasm is very electron dense and contains no bundles of filaments (unlike SPC and PRC I–II; Fig. 7). Furthermore, the euchromatin and heterochromatin within the nucleus of PRC IV is much more electron dense than in the other retinal cells (Fig. 6A,C and Fig. 7A), more closely resembling that of the dense photoreceptor in the gastropod *Bulla* sp. (Jacklet and Colquhoun, 1983). A similar photoreceptor cell was observed in the eye of *Ilyanassa* sp., which shows the same dense cytoplasm, narrowing of the cell body towards the pigmented region and irregular small microvilli as seen in PRC IV; however, unlike PRC IV, it lacks electron-lucent photic vesicles (Gibson, 1984; Fig. 8; Movie 2; Fig. 5 and Fig. 6F).

Although some clues as to cellular function are given by structural features as discussed above, further work is required to investigate what implications there are for the four strombid photoreceptors on visual processing. Electrophysiological investigations into the neural mechanisms of the *C. luhuanus* visual system indicated that photic stimulation triggers highly complex neural interactions, involving excitation, inhibition and oscillatory ‘off’ activity, unlike gastropods *Otala* and *Cornu* spp., which exhibited only excitation activity (Goldman and Hermann, 1967; Gillary, 1970, 1974, 1977). Gillary (1974) suggested that some processing of neural information occurs in the retina, similar to vertebrate retinae which act as complex filters to transfer specific information (including motion, contrast, colour and resolution) about images to the brain in parallel via different classes of ganglion cells (Wässle, 2004; Knudsen, 2020). By contrast, cephalopods do not possess ganglion cells in their retinas (Yamamoto et al., 1965), although some visual processing is thought to take place in the retina (Chung and Marshall, 2017). This is contrary to previous studies, which suggested that visual information processing is solely undertaken in the large optic lobe of the cephalopod brain (Young, 1962b). Therefore, visual processing in the strombid retina, as suggested by Gillary (1974) and this study, as well as the diversity of photoreceptor cell types in the retina, indicate that visual processing in strombids is different to that of cephalopods. The convergent evolution of large, high-acuity camera-type eyes in cephalopod and conch snails, yet non-convergent visual processing, makes the strombids an interesting subject for understanding the hierarchical steps in visual data processing and the evolution of vision in molluscs.

### Conclusions

This study provides behavioural evidence that the strombid gastropod *C. luhuanus* has high contrast sensitivity and high visual acuity, demonstrating that this species can respond to a Michelson contrast of ∼0.07 and differences as small as 1.06 deg in the visual field. The estimated spatial resolution from behavioural data is strongly supported by an estimate of 1.04 deg from anatomical data. This is the most acute vision described for any non-predatory gastropod and supports previous estimates based on morphological data, demonstrating the value of integrating morphological and behavioural approaches when studying visual function. Withdrawal responses by *C. luhuanus* to expanding stimuli suggest that high visual acuity and sensitivity is likely to play a vital role in early predator detection in this species; however, this resolution seems far superior to that required for this task when compared to visual acuity in other gastropods, and it is probable that high spatial resolution also underpins other behaviours in strombids.

New techniques (SBF-SEM, in conjunction with TEM) reveal six kinds of retinal cells within the *C. luhuanus* retina: a supportive cell, a ganglion cell, and four photoreceptor type cells I–IV. Two of these cells, the ganglion cell and the fourth photoreceptor cell are newly discovered and described for the first time in this study. These data provide new insights into cell functions and widens our understanding of the complexity of the retina structure in strombids. These findings suggest that strombids have a more complex retina compared to those within cephalopods and other gastropod groups, suggesting differences in the way visual information is processed among molluscs. However, the fact that these new techniques have identified a higher cell diversity in conch snails may also suggest that gastropods have more complex retinas in general than is currently known.

## Supplementary Material

Supplementary information
