# Supplementary material for: The marine gastropod Conomurex luhuanus (Strombidae) has high-resolution spatial vision and eyes with complex retinas
Source: J Exp Biol. 2022 Aug 26;225(16):jeb243927. doi: 10.1242/jeb.243927 (PMC9482149; doi:10.1242/jeb.243927)
Supplement: Supplementary information [file jexbio-225-243927-s1.pdf]

Figure S1

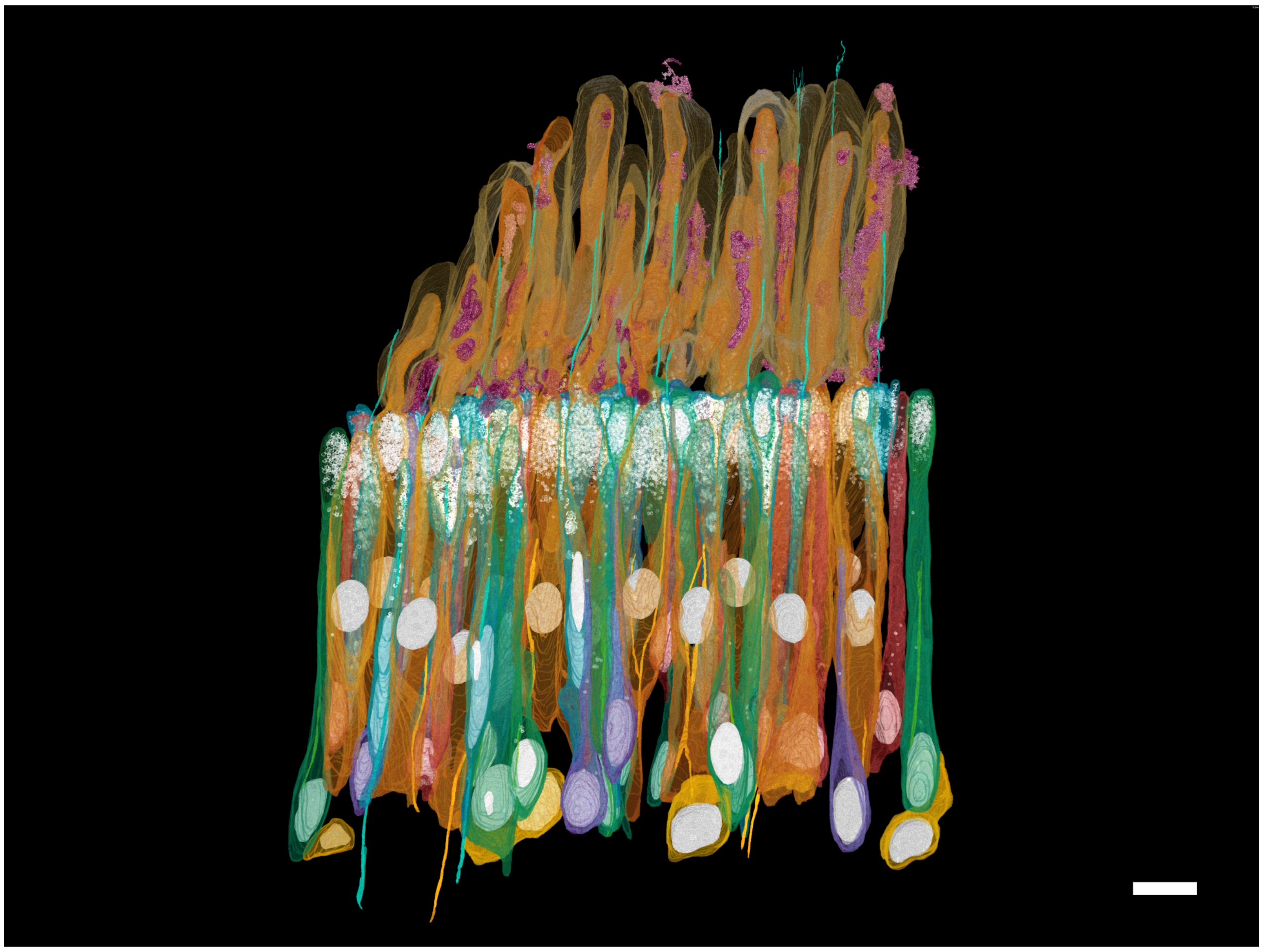

Fig. S1. Unedited image in support of Figure 5, showing retina structure of *Conomurex luhuanus* with cells segmented and reconstructed using SBF-SEM data: blue, SPC; orange, PRC I; green, PRC II; purple, PRC III; red, PRC IV; yellow, ganglion cell; pink, phagocytic activity. Nuclei and pigment are highlighted in white. Abbreviations: c, capsule; cc, cytoplasmic core (of PRC I distal segment); ds, distal segments; mv, microvilli; n, nucleus; np, neuropile; on, optic nerve; pc, pigment cluster; pg, pigment granule; pv, photic vesicles; tf, tonofilaments. Scale bar = 20 μm.

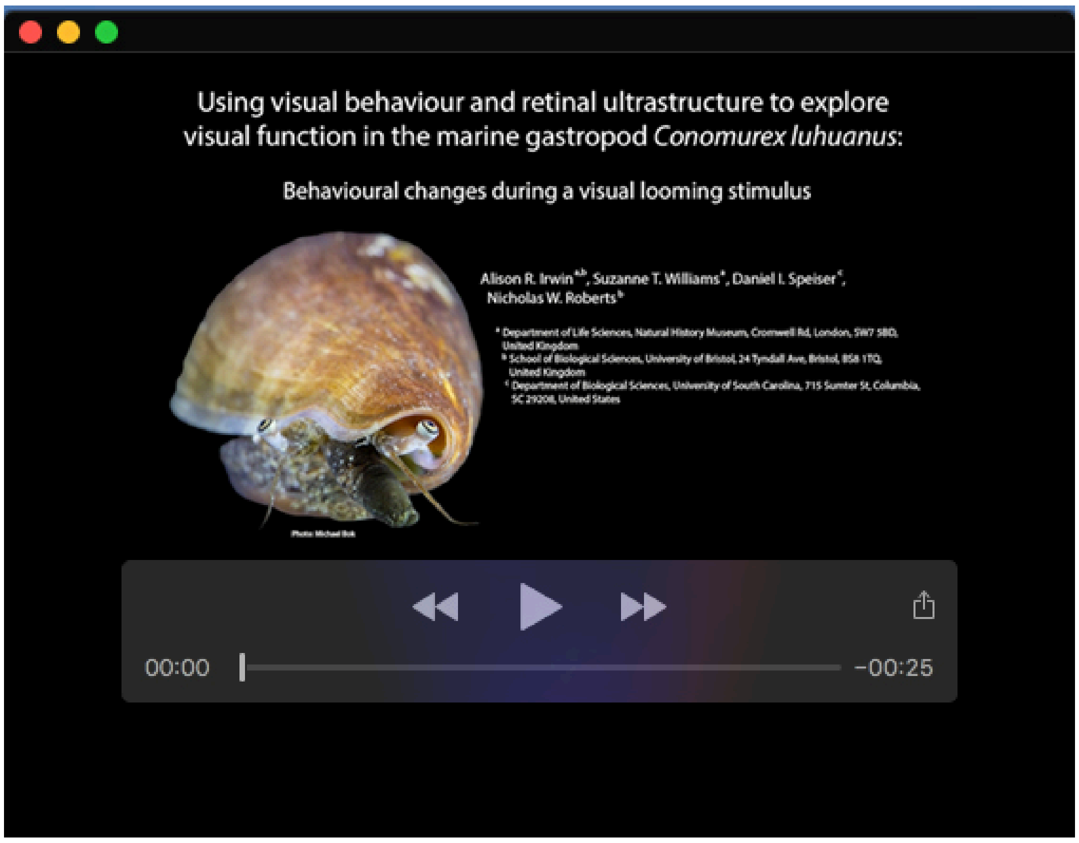

Movie 1. Video of *Conomurex luhuanus* displaying changes in behaviour in response to a looming stimulus. See Table 1 for full descriptions of behavioural responses identified in the video.

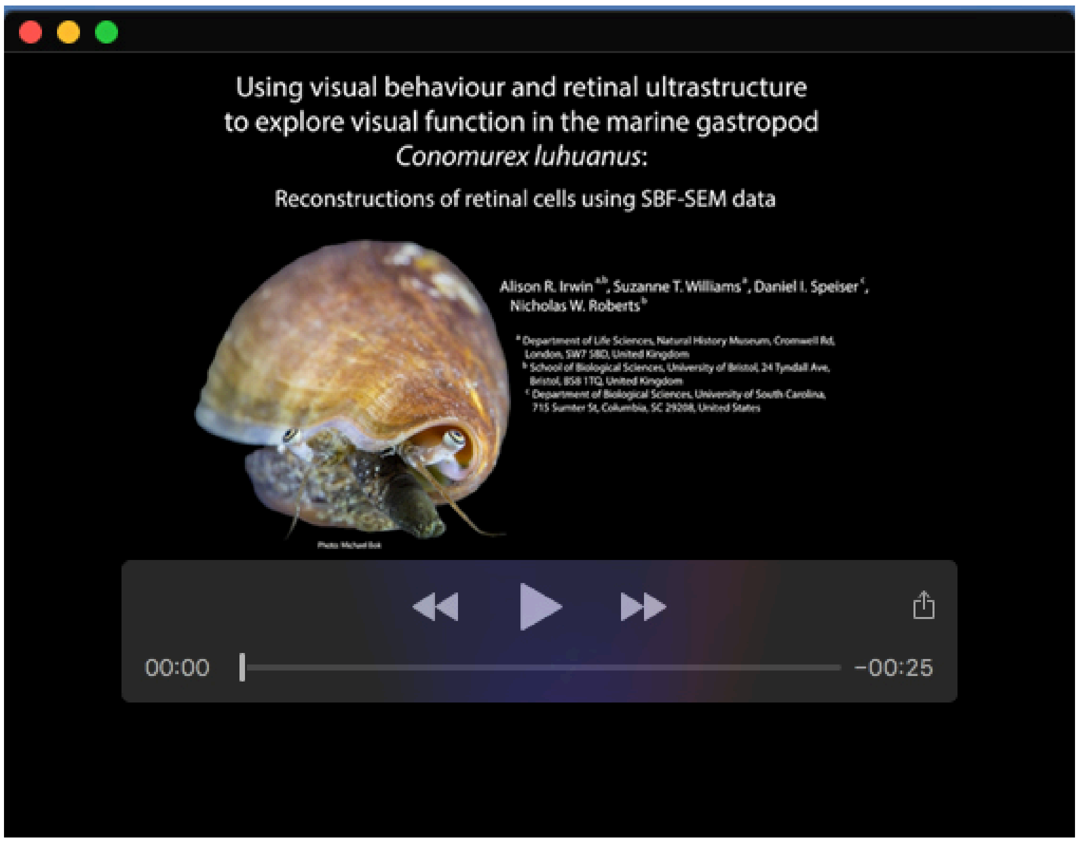

Movie 2. Three-dimensional volume reconstruction of cells in the *Conomurex luhuanus* retina, segmented from SBF-SEM data via VGStudio Max v. 2.2. Scale bar = 10 µm.
